# Central chemoreflex activation induces sympatho‐excitation without altering static or dynamic baroreflex function in normal rats

**DOI:** 10.14814/phy2.13406

**Published:** 2017-09-12

**Authors:** Keita Saku, Takeshi Tohyama, Masako Shinoda, Takuya Kishi, Kazuya Hosokawa, Takuya Nishikawa, Yasuhiro Oga, Takafumi Sakamoto, Hiroyuki Tsutsui, Tadayoshi Miyamoto, Kenji Sunagawa

**Affiliations:** ^1^ Department of Advanced Risk Stratification for Cardiovascular Diseases Center for Disruptive Cardiovascular Medicine Kyushu University Fukuoka Japan; ^2^ Department of Cardiovascular Medicine Graduate School of Medical Sciences Kyushu University Fukuoka Japan; ^3^ Graduate School of Health Sciences Morinomiya University of Medical Sciences Osaka Japan; ^4^ Department of Therapeutic Regulation of Cardiovascular Homeostasis Center for Disruptive Cardiovascular Medicine Kyushu University Fukuoka Japan

**Keywords:** Arterial pressure regulation, carotid sinus baroreflex, central chemoreflex, sympathetic nerve activity

## Abstract

Central chemoreflex activation induces sympatho‐excitation. However, how central chemoreflex interacts with baroreflex function remains unknown. This study aimed to examine the impact of central chemoreflex on the dynamic as well as static baroreflex functions under open‐loop conditions. In 15 anesthetized, vagotomized Sprague‐Dawley rats, we isolated bilateral carotid sinuses and controlled intra‐sinus pressure (CSP). We then recorded sympathetic nerve activity (SNA) at the celiac ganglia, and activated central chemoreflex by a gas mixture containing various concentrations of CO_2_. Under the baroreflex open‐loop condition (CSP = 100 mmHg), central chemoreflex activation linearly increased SNA and arterial pressure (AP). To examine the static baroreflex function, we increased CSP stepwise from 60 to 170 mmHg and measured steady‐state SNA responses to CSP (mechanoneural arc), and AP responses to SNA (neuromechanical arc). Central chemoreflex activation by inhaling 3% CO_2_ significantly increased SNA irrespective of CSP, indicating resetting of the mechanoneural arc, but did not change the neuromechanical arc. As a result, central chemoreflex activation did not change baroreflex maximum total loop gain significantly (−1.29 ± 0.27 vs. −1.68 ± 0.74, N.S.). To examine the dynamic baroreflex function, we randomly perturbed CSP and estimated transfer functions from 0.01 to 1.0 Hz. The transfer function of the mechanoneural arc approximated a high‐pass filter, while those of the neuromechanical arc and total (CSP‐AP relationship) arcs approximated a low‐pass filter. In conclusion, central chemoreflex activation did not alter the transfer function of the mechanoneural, neuromechanical, or total arcs. Central chemoreflex modifies hemodynamics via sympatho‐excitation without compromising dynamic or static baroreflex AP buffering function.

## Introduction

Animals including humans have evolved complex control mechanisms of circulatory and respiratory systems. They operate collaboratively to maintain appropriate levels of oxygen uptake, carbon dioxide (CO_2_) emission, pH and metabolism in the peripheral organs and tissues. The chemoreflexes are important modulators of ventilation. The peripheral chemoreceptors located in the carotid bodies respond primarily to hypoxia. Central chemoreceptors are located in the brain stem region, and respond primarily to changes in arterial CO_2_ content (PaCO_2_) (Berger et al. 1977; Bianchi et al. 1995; Dejours 1962; Kara et al. 2003). Specific brainstem neurons (including retrotrapezoid nucleus and serotonergic neurons) are activated by increase in PaCO_2_ and stimulate breathing (Guyenet and Bayliss 2015; Guyenet 2014). Under physiological conditions, central chemoreflex via central chemoreceptors also changes sympathetic nerve activity (SNA) and elicits teleologically sound cardiovascular responses (Somers et al. 1989). In contrary, under pathological conditions such as heart failure and hypertension, augmented central chemoreflex induces excessive sympatho‐excitation leading to poor prognosis (Giannoni et al. 2009; Li et al. 2016; Narkiewicz et al. 1999). Therefore, central chemoreflex is an important mechanism that activates SNA and contributes to circulatory homeostasis and cardiovascular pathophysiology.

The baroreflex operates as a negative feedback system and regulates SNA tightly to stabilize arterial pressure (AP). To assess the impact of central chemoreflex on sympathetic nervous system and circulatory regulation, the interaction between central chemoreflex and baroreflex is a key issue to be solved. The sympathetic baroreflex can be divided into two subsystems by baroreflex open‐loop analysis: a mechanoneural arc that describes how the baroreceptor pressure changes SNA, and a neuromechanical arc that describes how the SNA changes AP (Sato et al. 1999). Thus, an operation of the baroreflex obscures the isolated central chemoreflex activation effects on SNA and AP. In addition, identification of the open‐loop dynamic function is a prerequisite to understand how quickly and how stably baroreflex operates to regulate AP (Ikeda et al. 1996). Several investigators have reported the effect of central chemoreflex on the static baroreflex function. (Bristow et al. (1971) and Henry et al. (1998) have demonstrated a downward resetting of baroreflex control of R‐R interval (to higher heart rates) during activation of central chemoreflex. More recently, Simmons et al. (2007) have reported that mild activation of central chemoreflex does not appear to alter barorelfex controlled heart rate and SNA. However, those methods are incomplete as they do not allow the assessment of baroreflex control of AP. To identify the baroreflex function of AP regulation over the entire input range and the isolated impact of central chemoreflex activation on SNA and static and dynamic baroreflex function, the baroreflex open‐loop analysis is required.

With this background, we investigated the impact of central chemoreflex activation on static and dynamic baroreflex controls of SNA and AP regulation by performing baroreflex open‐loop analysis in normal rats.

## Materials and Methods

### Animals and Surgical preparations

Experiments and animal care were approved by the Committee on Ethics of Animal Experiment, Kyushu University Graduate School of Medical Sciences, and performed in accordance with the 8th Edition of the Guide for the Care and Use of Laboratory Animals published by National Academy Press in 2011.

Fifteen male Sprague–Dawley rats weighing 487 ± 34 g (Japan SLC, Inc., Hamamatsu, Japan) were used. A rat was anesthetized by an intraperitoneal injection (2 mL/kg) of a mixture of *α*‐chloralose (40 mg/mL) and urethane (250 mg/mL), and ventilated mechanically. The depth of anesthesia was maintained by infusing a 20‐fold dilution of the above anesthetic mixture from the right femoral vein (2–3 mL/kg per hour). AP was measured using a high‐fidelity pressure transducer (Millar Instruments; Houston, TX) inserted into the right femoral artery. An arterial catheter was inserted into the left femoral artery for blood sampling. Body temperature was maintained by a heating pad at approximately 38°C. We recorded SNA at the splanchnic sympathetic nerve by the method described previously (Saku et al. 2014). To quantify SNA, preamplified nerve signals were band‐pass filtered at 150–1000 Hz, and then full‐wave rectified and low‐pass filtered at a cut‐off frequency of 30 Hz using analog circuits. Pancuronium bromide (0.4 mg/kg per hour) was infused continuously to prevent electrical contamination of SNA from muscular activity. At the end of the experiment, a bolus injection of the ganglionic blocker hexamethonium (60 mg/kg) was given to confirm zero level of SNA and to detect the noise level of recorded nerve activity. To open the carotid baroreflex loop, bilateral carotid sinus baroreceptor regions were isolated from systemic circulation by a modification of previously reported method (Sato et al. 1999). Briefly, a 5–0 silk thread was passed between the external and internal carotid arteries, and the external carotid artery was ligated close to the carotid bifurcation. The internal carotid artery was embolized with three to five steel balls (0.8 mm in diameter; Tsubaki Nakashima, Nara, Japan) injected from the common carotid artery. The isolated carotid sinuses were filled with saline through catheters inserted into the common carotid arteries. Carotid sinus pressure (CSP) was controlled using a servo‐controlled piston pump. We also ligated bilateral external carotid arteries with 5–0 silk at a level 4–6 mm distal to the carotid bifurcation to shut down the blood flow of carotid body and to minimize the effect of peripheral chemoreflex. Heparin sodium (100 U/kg) was injected intravenously to prevent blood coagulation. Bilateral aortic depressor and vagal nerves were cut at the neck.

Central chemoreflex was activated by inhalation of a gas mixture of 40% oxygen and nitrogen containing various concentrations of CO_2_ (0, 3, 5%). In each protocol, we checked PaCO_2_.

### Protocols

After the surgical procedures were completed, we monitored responses of SNA and AP to CSP input for more than 30 min. After stabilization, CSP was matched to AP using a servo‐controlled piston pump and baseline hemodynamic data were recorded for 10 min.

#### Protocol 1: Impact of central chemoreflex activation on SNA and AP (*n* = 5)

To assess the relationship between central chemoreflex activation and SNA or AP under isolated condition, we controlled CSP at a constant pressure (100 mmHg) to abolish the pressure buffering effect of baroreflex. We administered CO_2_ gas mixture to activate central chemoreflex for 5 min and assessed the changes in SNA and AP. The CO_2_ content in the gas mixture was increased from 0 to 5% (0, 3 and 5%).

#### Protocol 2: Effect of central chemoreflex activation on baroreflex static function (*n* = 5)

To estimate the open‐loop static arterial baroreflex function, we increased CSP stepwise from 60 to 170 mmHg every 20 sec. Static function of arterial baroreflex subsystems were represented as wide‐range steady state relations between CSP and SNA (mechanoneural arc), SNA and AP (neuromechanical arc), and CSP and AP (total arc). We compared each arc at baseline and under central chemoreflex activation. Central chemoreflex activation was induced by inhalation of 3% CO_2_.

#### Protocol 3: Effect of central chemoreflex activation on baroreflex dynamic function (*n* = 5)

To obtain the open‐loop dynamic arterial baroreflex function, we altered CSP by 20 mmHg above or below the operating AP every 500 msec according to a random binary sequence for 10–20 min, and obtained the time series data of the CSP–SNA and SNA–AP relationships (Saku et al. 2014). We used 5‐min time series data for analysis. Then, we identified the transfer functions (H) of baroreflex mechanoneural (H_*CSP‐SNA*_), neuromechanical (H_*SNA‐AP*_) and total (H_*CSP‐AP*_) arcs, and compared the parameters between baseline and central chemoreflex activation. H obtained for each arc represents the dynamic function at operating point. They indicate how quickly the system works at the operating AP. Central chemoreflex activation was induced by inhalation of 3% CO_2_.

### Data analysis

Experimental time series data were recorded at the sampling frequency of 200 Hz using a 16 bit analog‐to‐digital converter (Power Lab 16/35, ADInstruments) and stored in a dedicated laboratory computer system. In protocol 1, SNA and AP measured at baseline and under central chemoreflex activation were averaged for the last 30 sec of each CO_2_ concentration. The SNA obtained were normalized by the averaged baseline value.

In protocol 2, to estimate the input–output relationship at steady state, AP and SNA were averaged over the last 10 sec for each stepwise CSP increase. Static characteristics of the arterial baroreflex mechanoneural arc (CSP–SNA relationship) and total arc (CSP–AP relationship) approximate an inverse sigmoidal curve, and are quantified using a four‐parameter logistic function as follows (Kent et al. 1972):

$$y=\frac{P_{1}}{1+\exp[P_{2}\left(x-P_{3}\right)]}+P_{4}$$

where x and y represent the input (CSP) and the output (SNA or AP) values, respectively; *P*1 is the response range of y; *P*2 is the slope coefficient; *P*3 is the midpoint of the sigmoid curve on x axis; and *P*4 is the minimum value of y. The maximum gain (G max) is −*P*1 × *P*2 /4 at *x* = *P*3. Static characteristics of the neuromechanical arc (SNA–AP relationship) approximate a straight line, and are quantified using linear regression as follows (Saku et al. 2014):

AP=a×SNA+b

where *a* and *b* represent the slope and intercept, respectively. The SNA obtained was normalized using the average integrated value across the period when CSP = 60 mmHg at baseline.

In protocol 3, the input–output data pairs were divided into 5 segments and processed with 50% overlapping bins of 1024 points each by a fast Fourier transform algorithm to identify transfer function. For each segment, the linear trend was subtracted and a Blackman–Harris window was applied. A fast Fourier transform was performed to obtain the spectra of the data segments. The ensemble averages of input [S*xx* (*f*)], output [S*yy* (*f*)] and cross‐spectral signal between input and output [S*yx* (*f*)] were estimated over the eight segments. Finally, the transfer function [H (*f*)] from input to output was calculated as follows (Marmarelis and Marmarelis 1978):

$$H\left(f\right)=\frac{S_{yx}\left(f\right)}{S_{xx}\left(f\right)}$$

The gain of transfer function of mechanoneural arc was normalized using the value of averaged dynamic gain below 0.03 Hz in each animal at baseline. We obtained the modulus [H (*f*)] and phase [*θ* (*f*)] of the transfer function using the following equations (Marmarelis and Marmarelis 1978).

$$\left|H\left(f\right)\right|=\sqrt{H_{Re}{\left(f\right)}^{2}+H_{Im}{\left(f\right)}^{2}}$$

$$\theta \left(f\right)=\mathrm{ta}n^{-1}\frac{H_{Im}\left(f\right)}{H_{Re}\left(f\right)}$$

where *H*_*Re*_ *(f)* is the real part and *H*_*Im*_ *(f)* is the imaginary part of *H(f)*. To quantify the linear dependence between the input and output signals in the frequency domain, a magnitude‐squared coherence function [Coh (*f*)] was calculated as follows (Marmarelis and Marmarelis 1978).

$$\mathrm{Coh}\left(f\right)=\frac{|S_{yx}\left(f\right){|}^{2}}{S_{xx}\left(f\right)S_{yy}\left(f\right)}$$

The coherence value ranges from zero to unity. Unity coherence indicates complete linear dependence between the input and output signals, whereas zero coherence indicates total independence between the two signals. To help understand the transfer function in dynamic analysis of baroreflex, the step responses calculated from the transfer function are also presented. The system impulse response was derived by inverse Fourier transform of H(*f*). The time integral of the impulse response yields the step response as follows:

$$Y\left(t\right)=\sum_{k=0}^{t}h\left(k\right)$$

where h(*k*) is the impulse response obtained by inverse Fourier transform of the transfer function (H(*f)*); t is the time.

### Statistical analysis

In protocol 1, the effects of central chemoreflex activation on PaCO_2_, SNA, and AP were evaluated by either paired *t*‐test or one‐way repeated measures ANOVA with Bonferroni post hoc test. In protocol 2, the effects of central chemoreflex activation on the parameters of baroreflex mechanoneural, neuromechanical, and total arcs, as well as on the closed‐loop operating point were examined using paired *t*‐test. In protocol 3, to test the difference in baroreflex dynamic function between baseline and central chemoreflex activation, we obtained the gain and phase values at 0.01, 0.1, 0.5 and 0.75 Hz, and step response at 1 (mechanoneural arc only), 5, 10, and 30 sec in each animal. The differences in these values between two conditions were examined by paired *t*‐test. Differences were considered significant when *P *<* *0.05.

## Results

### Impact of central chemoreflex activation on sympathetic nerve activity and arterial pressure

**Figure 1:**
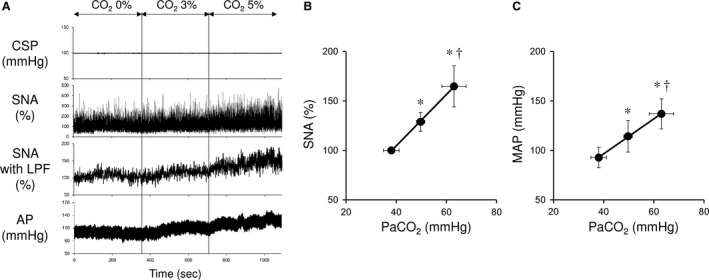
(A) Representative time series of sympathetic nerve activity (SNA) and arterial pressure (AP) under central chemoreflex activation from one rat. Central chemoreflex activation was induced by inhalation of 3 and 5% CO_2_ gas mixture. Changes in sympathetic nerve activity (B) and mean arterial pressure (C) under central chemoreflex activation induced by inhalation of a gas mixture containing 3% and 5% CO_2_. The data were obtained from five rats. Both the PaCO_2_–SNA (SNA = 2.60 × PaCO_2_ + 0.65, *r* ^2 ^= 0.9994) and PaCO_2_–mean AP relationships (AP = 1.77 × PaCO_2_ + 25.77, *r* ^2^ = 0.9995) show linearity. Changes in PaCO_2_ were shown in Table 1. CSP, carotid sinus pressure; SNA, sympathetic nerve activity; SNA with LPF, sympathetic nerve activity with low‐pass filter (cutoff frequency; 0.5 Hz); AP, arterial pressure; MAP, mean arterial pressure; PaCO_2_, partial pressure of carbon dioxide in arterial blood. Data are expressed as mean ± SD (*n* = 5). *P <* *0.05 versus baseline. ^†^ *P *<* *0.05 versus 3% CO_2_.

As summarized in Table 1, central chemoreflex activation by inhalation of CO_2_ gas mixture significantly increased PaCO_2_. Figure 1A shows typical time series of AP and SNA in response to central chemoreflex activation in one rat. Under central chemoreflex activation, SNA increased and reached a steady state in 1–2 min. AP also increased accompanying the SNA elevation. Figure 1B and C illustrate SNA and mean AP (MAP) in response to central chemoreflex activation averaged over five rats. At 3% and 5% CO_2_, SNA increased by 28.9 ± 9.6 and 64.8 ± 20.8%, respectively, from baseline (0% CO_2_). Both the PaCO_2_–SNA and PaCO_2_–AP relationships increased linearly.

**Table 1:** Arterial blood gas analysis

	Baseline	3% CO_2_	5% CO_2_
PaCO_2,_ mmHg	38.0 ± 3.1	49.7 ± 1.7^a^	63.0 ± 4.8^a^^,^ b
PaO_2,_ mmHg	191 ± 8	200 ± 12	190 ± 15
pH	7.46 ± 0.05	7.40 ± 0.07^a^	7.35 ± 0.04^a^

Changes in arterial blood gas averaged over five rats in protocol 1 among three conditions: baseline, 3% and 5% CO_2_ gas mixture. The gas mixture contained 40% oxygen and nitrogen with various concentrations of CO_2_ (0, 3, 5%). PaCO_2_, partial pressure of carbon dioxide in arterial blood; PaO_2_, partial pressure of oxygen in arterial blood. Data are expressed as mean ± SD.

a *P *<* *0.05 versus baseline.

b *P *<* *0.05 versus 3% CO_2_.

### Effects of central chemoreflex activation on static baroreflex function

**Figure 2:**
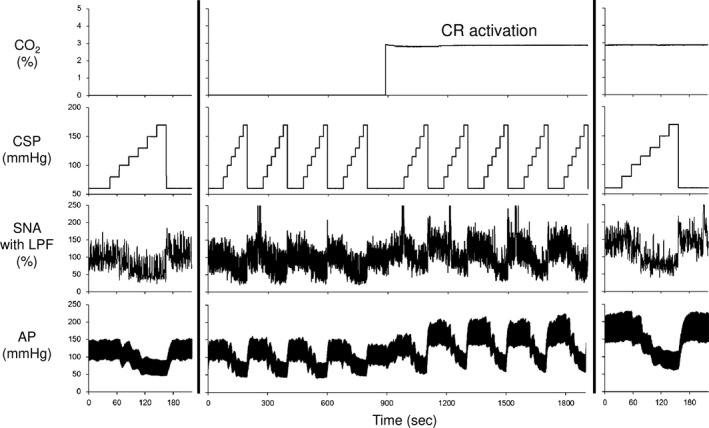
Typical time series of carotid sinus pressure (CSP), sympathetic nerve activity (SNA) and arterial pressure (AP) at baseline and after under central chemoreflex (CR) activation from one rat in protocol 2. Data were resampled at 50 Hz. SNA and AP decreased in response to stepwise increment in CSP both at baseline and under central chemoreflex activation. Central chemoreflex activation further increased SNA and AP at each level of CSP. CO_2_, concentration of CO_2_ (%) in gas mixture measured by gas analyzer; CSP, carotid sinus pressure; SNA with LPF, sympathetic nerve activity with low‐pass filter (cutoff frequency; 0.5 Hz); AP, arterial pressure.

**Figure 3:**
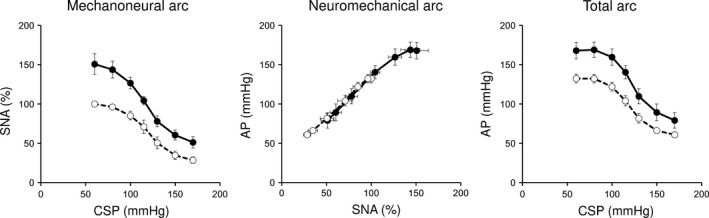
Mean baroreflex mechanoneural, neuromechanical and total arcs at baseline (dashed line with open circles) and under central chemoreflex activation (solid line with closed circles) averaged over five rats in protocol 2. Central chemoreflex activation shifted the mechanoneural arc to higher SNA, but did not change the neuromechanical arc. SNA, sympathetic nerve activity; CSP, carotid sinus pressure; AP, arterial pressure. Data are expressed as mean ± SEM.

Figure 2 shows representative recordings of CSP, SNA, and AP at baseline and under central chemoreflex activation in one rat. Inhalation of 3% CO_2_ significantly increased PaCO_2_ (baseline: 41.3 ± 0.6, central chemoreflex activation: 51.3 ± 2.3 mmHg, *p *<* *0.05). Stepwise increase in CSP (from 60 to 170 mmHg, 20 sec/step) resulted in sigmoidal decreases of SNA and AP, while central chemoreflex activation increased SNA and AP at every CSP increment. Figure 3 shows the baroreflex mechanoneural, neuromechanical, and total arcs at baseline and under central chemoreflex activation averaged over five rats. Under central chemoreflex activation, the baroreflex mechanoneural arc shifted upward while preserving a sigmoidal relationship. In the mechanoneural arc, the response range of SNA (*P*_1_), the coefficient of gain (*P*_2_), and midpoint of the operating range (*P*_3_) did not differ between baseline and central chemoreflex activation, while minimum SNA (*P*_4_) increased significantly from 24.9 ± 8.0 to 46.4 ± 13.9% (*P *<* *0.05), suggesting that central chemoreflex activation induced a parallel upward shift of the baroreflex mechanoneural arc (Table 2). The neuromechanical arc showed a linear relationship between SNA and AP (Fig. 3). In the neuromechanical arc, central chemoreflex activation did not alter any of the baroreflex parameters (Table 2). In the total arc, the response range of AP (*P*_1_), the coefficient of gain (*P*_2_) and midpoint of the operating range (*P*_3_) did not differ between baseline and central chemoreflex activation, while minimum AP (*P*_4_) increased significantly from 52.3 ± 16.6 to 74.0 ± 11.3 mmHg (*P *<* *0.05). Both maximum gain (baseline: −1.29 ± 0.27, central chemoreflex activation: −1.68 ± 0.74) and gain at operating point (baseline: −1.05 ± 0.34, central chemoreflex activation: −1.55 ± 0.77) did not differ significantly between baseline and central chemoreflex activation. These data suggest that central chemoreflex activation induced a parallel upward shift of baroreflex mechanoneural arc without significant changing the pressure buffering function of the baroreflex (Table 2).

**Table 2:** Effects of central chemoreflex activation on the parameters of baroreflex mechanoneural, neuromechanical and total arcs

	Baseline	CR activation
Mechanoneural arc (CSP–SNA relationship)		
*P*1, %	79.0 ± 9.5	101.2 ± 25.0
*P*2, %/mmHg	0.063 ± 0.017	0.057 ± 0.011
*P*3, mmHg	126.2 ± 16.1	123.5 ± 15.2
*P*4, %	24.9 ± 8.0	46.4 ± 13.9^a^
Coefficient of determination (R^2^)	0.990 ± 0.010	0.990 ± 0.007
Neuromechanical arc (SNA–AP relationship)		
*a*, mmHg/%	1.00 ± 0.20	1.02 ± 0.29
*b*, mmHg	37.5 ± 16.2	28.7 ± 35.1
Coefficient of determination (R^2^)	0.986 ± 0.013	0.981 ± 0.022
Total arc (CSP–AP relationship)		
*P*1, mmHg	84.2 ± 28.2	94.7 ± 16.4
*P*2	0.067 ± 0.021	0.070 ± 0.021
*P*3, mmHg	127.8 ± 9.8	130.6 ± 7.0
*P*4, mmHg	52.3 ± 16.6	74.0 ± 11.3^a^
G max	−1.29 ± 0.27	−1.68 ± 0.74
Gain at operating point	−1.05 ± 0.34	−1.55 ± 0.77
Coefficient of determination (R^2^)	0.996 ± 0.003	0.997 ± 0.003

Each value is averaged over five rats in protocol 2. The coefficient of determination (R^2^) as an indicator of fitting accuracy in each arc is shown. Data are expressed as means ± SD. CR activation, central chemoreflex activation; CSP, carotid sinus pressure; SNA, sympathetic nerve activity; AP, arterial pressure; *P*1, response range; *P*2, coefficient of gain; *P*3, midpoint of the operating range; *P*4, minimum SNA or AP; *a*, slope; *b*, intercept; G max, maximum gain (=−*P*1 × *P*2 /4).

a *p *<* *0.05 versus baseline.

### Effects of central chemoreflex activation on dynamic baroreflex function

**Figure 4:**
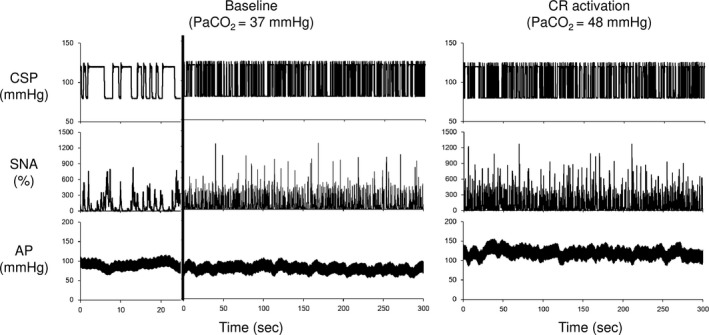
Representative time series of sympathetic nerve activity (SNA) and arterial pressure (AP) under carotid sinus pressure (CSP) perturbation from one rat in protocol 3. Data were resampled at 50 Hz. CSP was perturbed according to binary white noise sequences. Inhalation of 3% CO_2_ gas mixture increased PaCO_2_ from 37 mmHg to 48 mmHg in this rat. CSP perturbation changed SNA and AP. Under central chemoreflex (CR) activation, SNA and AP increased at each CSP level. CSP, carotid sinus pressure; SNA, sympathetic nerve activity; AP, arterial pressure.

**Figure 5:**
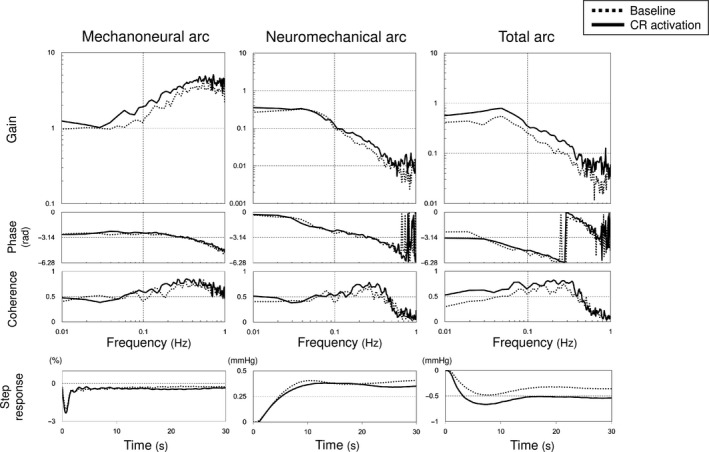
Transfer functions (H) of mechanoneural, neuromechanical and total arcs obtained at baseline (dashed line) and under central chemoreflex (CR) activation (solid line). Mean gain, phase (radian), coherence and step response averaged over five rats are shown. The error band of each graph is not shown for visibility. The gain of transfer function in mechanoneural arc was normalized by the mean dynamic gain below 0.03 Hz at baseline.

Illustrated in Figure 4 are typical time series of CSP, SNA, and AP under CSP perturbation at baseline and with central chemoreflex activation in one rat. CSP was perturbed according to a binary white noise sequence (mean AP ± 20 mmHg). CSP perturbation resulted in SNA and AP changes via arterial baroreflex. Figure 5 shows the averaged transfer functions of H_*CSP‐SNA*_ (baroreflex mechanoneural arc), H_*SNA‐AP*_ (baroreflex neuromechanical arc) and H_*CSP‐AP*_ (baroreflex total arc) at baseline and under central chemoreflex activation averaged over five rats. In the baroreflex mechanoneural arc, the gain increased with frequency reflecting its derivative characteristics. The phase approached –*π* radians at the lowest frequency, reflecting an out‐of‐phase relationship, that is, negative feedback feature, for CSP–SNA. In the baroreflex neuromechanical arc, the dynamic gain decreased with frequency, indicating low‐pass filter characteristics. The phase approached zero radians at the lowest frequency, reflecting an in‐phase relationship and that a rise in SNA increased AP. In the baroreflex total arc, the dynamic gain showed low‐pass filter characteristics and an out‐of‐phase relationship, indicating that a rise in CSP decreased AP. Table 3 summarizes the gain and phase values of the transfer functions of baroreflex at baseline and under central chemoreflex activation. In both mechanoneural and neuromechanical arcs of the baroreflex, the gain and phase at 0.01, 0.1, 0.5, and 0.75 Hz did not differ significantly between baseline and central chemoreflex activation, suggesting that central chemoreflex activation did not alter the dynamic function of the baroreflex subsystems. As a result, the step responses back‐calculated from transfer functions of baroreflex mechanoneural and neuromechanical arcs were not significantly different between the two conditions. In the total arc also, the gain and phase at 0.01, 0.1, 0.5 and 0.75 Hz, as well as step response, did not differ significantly between baseline and central chemoreflex activation, suggesting that central chemoreflex activation did not alter the dynamic pressure buffering function of the baroreflex.

**Table 3:** Effects of central chemoreflex activation on the transfer functions of baroreflex mechanoneural, neuromechanical and total arcs

	Mechanoneural arc (%/mmHg)		Neuromechanical arc (mmHg/%)		Total arc (mmHg/mmHg)	
	Baseline	CR activation	Baseline	CR activation	Baseline	CR activation
Gain						
0.01 Hz	0.97 ± 0.06	1.25 ± 0.36	0.27 ± 0.18	0.35 ± 0.07	0.41 ± 0.13	0.56 ± 0.18
0.1 Hz	1.19 ± 0.27	1.91 ± 0.27	0.10 ± 0.04	0.13 ± 0.06	0.26 ± 0.09	0.36 ± 0.15
0.5 Hz	3.54 ± 0.25	4.67 ± 0.27	0.011 ± 0.005	0.015 ± 0.01	0.05 ± 0.02	0.08 ± 0.05
0.75 Hz	3.31 ± 0.47	3.85 ± 0.48	0.005 ± 0.004	0.006 ± 0.01	0.02 ± 0.01	0.07 ± 0.04
Phase (radian)						
0.01 Hz	−2.72 ± 0.39	−2.77 ± 0.14	−0.35 ± 0.37	−0.29 ± 0.13	−2.45 ± 0.29	−3.20 ± 0.42
0.1 Hz	−2.61 ± 0.09	−2.57 ± 0.08	−2.64 ± 0.13	−2.36 ± 0.18	−4.93 ± 0.14	−4.74 ± 0.18
0.5 Hz	−3.79 ± 0.11	−3.55 ± 0.21	−4.11 ± 0.17	−4.16 ± 0.21	−1.79 ± 0.17	−1.32 ± 0.37
0.75 Hz	−4.50 ± 0.16	−4.19 ± 0.26	−4.62 ± 0.48	−6.05 ± 0.74	−3.65 ± 0.56	−3.24 ± 0.43

Each value is averaged over five rats in protocol 3. No significant differences were detected between baseline and central chemoreflex (CR) activation for the baroreflex mechanoneural, neuromechanical and total arcs. The gain of transfer function in mechanoneural arc was normalized by the mean dynamic gain below 0.03 Hz at baseline. Data are expressed as means ± SEM. Mechanoneural arc, CSP–SNA relationship; Neuromechanical arc, SNA–AP relationship; Total arc, CSP–AP relationship; CSP, carotid sinus pressure; SNA, sympathetic nerve activity; AP, arterial pressure.

## Discussion

We investigated the impact of central chemoreflex activation on the static and dynamic functions of the arterial baroreflex. The major findings of this study are: (1) Central chemoreflex activation increases SNA almost linearly under baroreflex open‐loop condition; (2) Central chemoreflex activation induces upward resetting of the baroreflex mechanoneural arc, while preserving the pressure buffering function of the baroreflex; and (3) Central chemoreflex activation does not alter baroreflex dynamic function.

### Impact of chemoreflex on sympathetic nerve activity

Central chemoreceptors respond primarily to changes in PaCO_2_ and alter ventilation (VE). Changes in ventilation alters PaCO_2_. Miyamoto et al. (2004) divided the central chemoreflex‐controlled respiratory system into central controller (PaCO_2_–VE relationship) and peripheral plant (VE–PaCO_2_ relationship), and established an equilibrium diagram of the integrated respiratory control system. Thus, central chemoreflex constitutes a powerful feedback control system mediated by PaCO_2_. Meanwhile, PaCO_2_ increase is also known to increase SNA (Somers et al. 1989). We observed in this study the isolated effect of central chemoreflex activation on SNA in baroreflex open‐loop condition. As shown in Figure 1B, increase in PaCO_2_ is accompanied by a linear increase in SNA (SNA = 2.60 × PaCO_2_ + 0.65, *r* ^2^ = 0.9994). Inhalation of 3% and 5% CO_2_ increased SNA by as much as 29% and 65%, respectively. Guyenet (2014) and Moreira et al. (2006) previously reported a linear relationship between PaCO_2_ and SNA in sino‐aortic denervated rats. Our results are consistent with their findings and suggest that central chemoreflex activation induces sympatho‐excitation with a linear relationship between PaCO_2_ and SNA, similar to respiratory activation.

### Effect of central chemoreflex activation on baroreflex function

The baroreflex is the most powerful regulator of SNA and controls arterial pressure. We divided the arterial baroreflex system into two subsystems. The mechanoneural arc (CSP–SNA relationship) approximates a reverse sigmoidal relationship, whereas the neuromechanical arc (SNA–AP relationship) a linear relationship. The two subsystems are connected in series; thereby forming a negative feedback system to attenuate AP disturbance (Sato et al. 1999). Without the knowledge of dynamic as well as static baroreflex open‐loop characteristics, there is no way of knowing how central chemoreflex activation affects the baroreflex regulation of AP. As shown in Figure 3, central chemoreflex activation shifted the mechanoneural arc upward, but did not change neuromechanical arc except for shifting the operating range. As shown in the parametric analysis (Table 2), central chemoreflex activation did not change the parameters of both subsystems except *P*_4_ of the mechanoneural arc. Our results are consistent of previous reports that demonstrated the effect of central chemoreflex activation on baroreflex‐controlled R‐R interval and SNA (Bristow et al. 1971; Henry et al. 1998; Simmons et al. 2007). Because we opened the baroreflex loop, we were able to obtain the CSP–SNA and SNA–AP relationships over a wide range, and elucidate the effect of central chemoreflex activation on the relationships. The increase in PaCO_2_ is well known to induce vasodilation that may counteract the increase in AP produced by sympathetic activation. Given the potent vasodilating effect of PaCO_2_ on systemic circulation (Fukuda et al. 1990), particularly the cerebral vascular bed (Hoskote et al. 2004; Kety and Schmidt 1948), CO_2_ gas inhalation could have shifted the function curve of neuromechanical arc downward and reduced AP. However, such phenomena were not observed in our study. We found that CO_2_ gas inhalation parallelly shifted the mechanoneural arc upward, while did not change the neuromechanical arc of baroreflex. Since AP is determined as the intersection between the two function curves, the increase in PaCO_2_ resulted in the elevation of AP. We speculate that exposure to higher concentration of CO_2_ gas loading or for a longer duration might have reduced peripheral vascular resistance and resulted in a fall in AP or downward shifting of neuromechanical arc.

**Figure 6:**
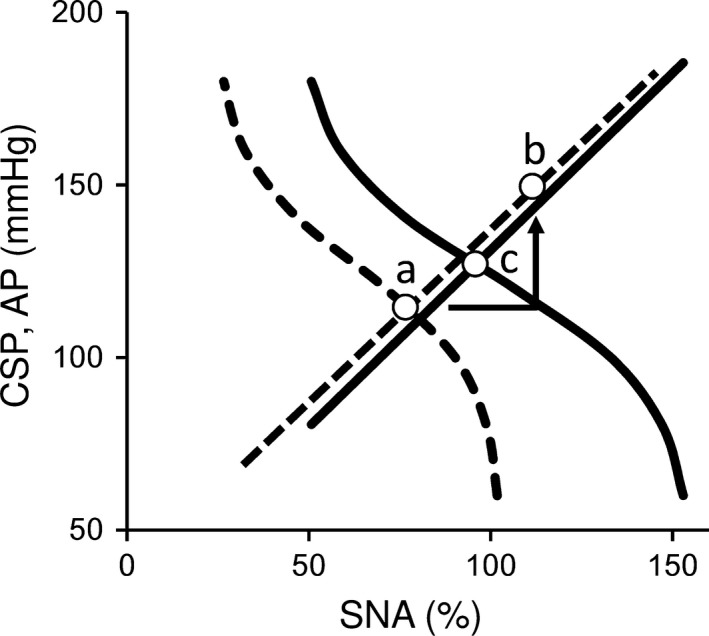
Baroreflex equilibrium diagram and the scheme of interaction between central chemoreflex activation and baroreflex. The dashed and solid lines indicate two subsystems of baroreflex function at baseline and under central chemoreflex activation, respectively. The operating point at baseline (a) is determined from the intersection of the mechanoneural and neuromechanical arcs. The absence of baroreflex feedback loop allows increase in AP to 146.3 mmHg and SNA to 114.8% (b). Since baroreflex forms a negative feedback loop, the operating point under central chemoreflex activation shifts to (c) at AP of 127.0 mmHg and SNA of 94.5%.

Figure 6 shows the equilibrium diagram with or without central chemoreflex activation using the averaged parameters of five rats. This diagram indicates that central chemoreflex activation significantly induces sympatho‐excitation and the absence of baroreflex feedback loop allows increase in AP to 146.3 mmHg and SNA to 114.8% (b). In the presence of baroreflex, the operating point, which is the intersection of the mechanoneural and neuromechanical arcs, shifts from *a* to *c* (increase in AP from 114.5 to 127.0 mmHg, and increase in SNA from 76.4 to 94.5%) under central chemoreflex activation. Therefore, intact baroreflex attenuates central chemoreflex activation induced sympatho‐excitation and AP elevation.

To elucidate the stability and variability in a biological system, it is necessary to identify not only static but also dynamic characteristics. Ikeda et al. (1996) reported that the high‐pass filter characteristics of the baroreflex mechanoneural arc complements the low‐pass filter neuromechanical arc and optimized dynamic AP regulation. As shown in Figure 5 and Tables 3 and 4, central chemoreflex activation did not alter the dynamic characteristics of both subsystems indicating that central chemoreflex activation does not change the stable and rapid response of the baroreflex in this setting. The fact that the central chemoreflex activation did not change dynamic gain of each arc (Fig. 5 and Table 3) is consistent with the finding of no significant change in static gain for each arc (Fig. 3 and Table 2).

**Table 4:** Effects of central chemoreflex activation on step responses of baroreflex mechanoneural, neuromechanical and total arcs

	Mechanoneural arc (%)		Neuromechanical arc (mmHg)		Total arc (mmHg)	
	Baseline	CR activation	Baseline	CR activation	Baseline	CR activation
Time (s)						
1	−1.65 ± 0.33	−1.83 ± 0.55	–	–	–	–
5	−0.51 ± 0.13	−0.40 ± 0.30	0.26 ± 0.09	0.24 ± 0.05	−0.42 ± 0.10	−0.62 ± 0.28
10	−0.42 ± 0.27	−0.36 ± 0.42	0.40 ± 0.12	0.36 ± 0.04	−0.45 ± 0.09	−0.62 ± 0.14
30	−0.25 ± 0.32	−0.35 ± 0.42	0.41 ± 0.14	0.35 ± 0.06	−0.36 ± 0.14	−0.54 ± 0.22

Each value is averaged over five rats in protocol 3. No significant differences in step responses at 1 (mechanoneural arc only), 5, 10, and 30 s were detected between baseline and central chemoreflex (CR) activation for the mechanoneural, neuromechanical and total arcs. Data are expressed as means ± SEM. Mechano‐neural arc, CSP‐SNA relationship; Neuromechanical arc, SNA–AP relationship; Total arc, CSP–AP relationship; CSP, carotid sinus pressure; SNA, sympathetic nerve activity; AP, arterial pressure.

**Figure 7:**
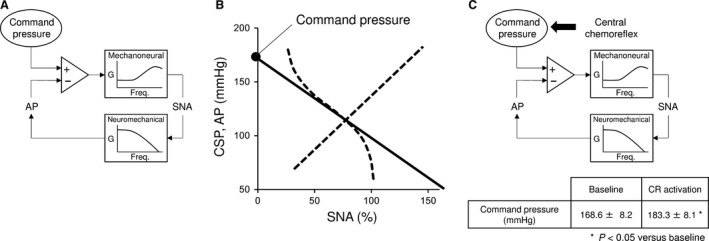
The effect of central chemoreflex activation on AP control system of the baroreflex. (A) The baroreflex mechanoneural arc regulates SNA by the product of gain (G) and difference between a given AP and command pressure. The baroreflex neuromechanical arc changes AP by SNA. Sato et al. (1999) proposed this framework. (B) The command pressure, in functional term, is the pressure that nullifies SNA on the tangential line drawn through the operating point of mechanoneural arc. It characterizes the mechanoneural arc around the operating point. The pressure axis intercept of linearized mechanoneural arc represents the command pressure. (C) Central chemoreflex (CR) activation increases the baroreflex command pressure (table), but does not change the dynamic characteristics of the baroreflex system. Thus, CR activation induces sympathetic‐excitation without compromising the pressure buffering function of the baroreflex.

From the control system point of view, baroreflex consists of a negative feedback loop as shown in Figure 7A. The mechanoneural arc regulates SNA by the difference between a given AP and command pressure. The baroreflex neuromechanical arc changes AP according to the SNA. The command pressure, in functional term, is the pressure that nullifies SNA on the tangential line drawn through the operating point of mechanoneural arc and is represented by the intercept of the pressure‐axis as shown in Figure 7B. Central chemoreflex activation increases the command pressure from 168.6 ± 8.2 to 183.3 ± 8.1 mmHg (Fig. 7C) and induces sympatho‐excitation without changing the shape of sigmoidal CSP–SNA relationship for the CSP range from 60 to 170 mmHg.

Taken together, we conclude that central chemoreflex activation, at least under acute CO_2_ loading condition, does not change the static and dynamic pressure buffering function of the arterial baroreflex. Resetting of the baroreflex mechanoneural arc by other reflex inputs has already been documented. Yamamoto et al. (2004) reported that muscle mechanoreflex reset the baroreflex mechanoneural arc upward and induced sympatho‐excitation. We previously reported that afferent vagal nerve stimulation reset baroreflex mechanoneural arc downward and evoked sympatho‐inhibitory effect (Saku et al. 2014). The present results are consistent with previous findings, suggesting that most afferent inputs that alter SNA integrate additively in the brain resulting in resetting of the baroreflex mechanoneural arc.

In addition, it is well known that augmented central chemoreflex sensitivity leads to sympatho‐excitation in several cardiovascular diseases such as heart failure and hypertension (Giannoni et al. 2009; Li et al. 2016; Narkiewicz et al. 1999). Further upward shifting of the mechanoneural arc and/or downward shifting of the neuromechanical arc may occur in heart failure and hypertension, and possibly deteriorates static and dynamic baroreflex functions by shifting the operating point. Thus, investigations focusing on how the central chemoreflex and baroreflex interact in such pathological conditions requires to be investigated in specific animal models of cardiovascular diseases.

### Physiological implication

Most biological systems are designed to be functional within a narrow pH range. This is a stringent requirement to keep homeostasis. Since the concentration of hydrogen ions in blood depends directly on that of carbon dioxide (Beaver et al. 1986), effective and appropriate removal of CO_2_ from blood is a prerequisite for homeostasis. Thus, it is teleologically sound that the circulatory system cooperates with the respiratory system to regulate cardiac output (CO) to effectively remove CO_2_ from the lung, while keeping AP constant to secure the perfusion of vital organs (Nobrega et al. 2014). We assumed that central chemoreflex senses PaCO_2_, delivers signals to the brain regarding the appropriate circulatory condition, and modifies SNA. Activation of SNA is known to increase cardiac systolic function, HR, arterial resistance and stressed volume (Funakoshi et al. 2014; Sakamoto et al. 2015). Especially under exercise condition, it is necessary to stimulate circulation and respiration against the increase in carbon dioxide production. In addition, the peripheral vascular resistance is reduced in exercise (Hagberg et al. 1985). In such situation, SNA could increase CO while maintaining AP. In other words, central chemoreflex allows carbon dioxide to be carried to the lung effectively by regulating the cardiovascular system without changing the AP stabilizing function.

### Limitation

There are several limitations in this study. First, we conducted this study under anesthetic condition. Since anesthesia strongly alters the autonomic function, the quantitative results measured under anesthesia may be different from those under conscious condition.

Second, we ligated bilateral common carotid arteries to isolate the carotid sinus regions, which might have reduced cerebral blood flow. Since a decrease in cerebral blood flow might reduce the diffusion of CO_2_ from the cerebrospinal fluid and brain extracellular fluid to the cerebral vessels, it might increase hydrogen ions in the central chemoreceptor regions (Xie et al. 2006) and affect the central chemoreflex sensitivity. Therefore, we cannot directly extrapolate our results to interpret the interaction between the central chemoreflex activation and baroreflex function under physiological conditions.

Third, the peripheral chemoreflex located in the carotid body, which mainly senses oxygen content in arterial blood, is also an important system to regulate respiration and sympathetic nervous system (Marshall 1994; Paton et al. 2013; Somers et al. 1989). In this study, to evaluate the isolated impact of central chemoreflex activation on baroreflex function, we diminished the peripheral chemoreflex interaction by ligating bilateral external carotid arteries that perfuse the carotid body and administered oxygen‐enriched gas (containing 40% O_2_) to the rats.

Fourth, although we focused on the effect of central chemoreflex on baroreflex function, baroreflex has a reciprocal direct effect on respiration. In a dog experiment, changes in the carotid sinus baroreceptor input altered spontaneous breathing (Brunner et al. 1982). Investigations of these reciprocal interactions between chemoreflex and baroreflex are needed to understand the integrative physiology of respiration and circulation.

## Conclusion

Central chemoreflex activation resets the baroreflex mechanoneural arc upward, but does not change baroreflex total loop gain significantly. Thus, central chemoreflex modifies hemodynamics via sympatho‐excitation without compromising the pressure buffering function of the baroreflex.

## Conflict of Interest

The authors declare no conflicts of interest, financial or otherwise.
